# Draft Genome Sequence of the Anoxygenic Phototrophic Bacterium *Rhodomicrobium* sp. Strain Az07, Isolated from a Brackish Canal

**DOI:** 10.1128/MRA.00585-21

**Published:** 2021-09-23

**Authors:** E. D. Bakhmutova, A. O. Izotova, S. V. Toshchakov, Z. B. Namsaraev, A. V. Komova

**Affiliations:** a National Research Center “Kurchatov Institute,” Moscow, Russia; Montana State University

## Abstract

*Rhodomicrobium* sp. strain Az07 was isolated from a brackish canal. The organism is more halotolerant than previously described species of the genus *Rhodomicrobium*. The Illumina MiSeq system was used to sequence the genome of the isolated strain. The assembly contains 3,291,400 bp, 106 contigs, and a GC content of 62.7%.

## ANNOUNCEMENT

To date, the genus *Rhodomicrobium* comprises 3 described species (Rhodomicrobium vannielii [1], Rhodomicrobium udaipurense [2], Rhodomicrobium lacus [3]). Representatives of this genus are phototrophic bacteria possessing long prosthecae and having a characteristic vegetative growth cycle. The type strain of the species *R. vannielii* was described as a mesophilic freshwater bacterium with a preference for slightly acidic pH. Nevertheless, *R. udaipurense* can tolerate NaCl up to 2%, and *R. lacus* is able to grow at a pH of up to 9.

*Rhodomicrobium* sp. strain Az07 was isolated from a brackish canal extending from the Akhtarsky Estuary (Azov Sea, Krasnodar Region, Russia). The mineralization in the canal was 7 g/liter. The sample of water and silt was used to inoculate agar shake dilutions with medium (4) modified in order to keep the mineralization of the sampling point. After a week-long incubation at 30°C at light (2,000 lx), brick-red colonies appeared in the anaerobic zone. The purity of the isolate obtained was confirmed using optical microscopy and 16S rRNA sequencing. The cells of strain Az07 had morphological features typical of *Rhodomicrobium*. The new strain was able to grow in medium containing up to 5% NaCl, with optimal growth at 0 to 1% NaCl.

The strain was grown on the modified liquid medium (4) for 3 days at 30°C in the light (2,000 lx). Genomic DNA was isolated from the cell cultures using the QIAamp DNA minikit following the manufacturer’s recommendations (Qiagen, Dusseldorf, Germany). A paired-end DNA library (average insert size, 342 bp) was constructed using the Nextera XT DNA library preparation kit for Illumina (Illumina, USA). The DNA library was sequenced using an Illumina MiSeq system with 150-bp paired-end reads.

The draft genome sequence of *Rhodomicrobium* sp. strain Az07 was constructed using the Shovill v. 1.1.0 pipeline (5). Error correction of the Illumina reads was conducted using Lighter (6), and the reads were trimmed using Trimmomatic (7). Overlapping and stitching of the paired-end reads were completed using FLASH v. 1.2.11 (8). A total of 2,893,049 reads were sequenced. These reads were assembled *de novo* using SPAdes v. 3.14.1 (9). The resulting assembly contained 106 contigs (*N*_50_, 168,394 bp). The draft genome sequence was annotated using the NCBI Prokaryotic Genome Annotation Pipeline (10). The coverage of the whole-genome sequence was 227.8×. The genome size is estimated to be 3,291,400 bp, with a GC content of 62.7%. Analysis of the assembly quality using CheckM (11) showed a high level of completeness (97.77%) and a low level of predicted contamination (0.13%).

The sequenced genome was compared to the closest genomes with ChunLab’s online average nucleotide identity (ANI) calculator, which uses the OrthoANIu algorithm (12). ANI analysis confirmed discrepancies with the genome of the closest neighbor. The *Rhodomicrobium* sp. strain Az07 genome has an ANI value of 86.55% compared with the genome of *Rhodomicrobium lacus* JA980 (GenBank assembly accession number GCA_003992725.1), with an average aligned length of 1,763,080 bp.

The total length is 3,291,400 bp, containing 3,032 coding sequences (CDSs) total, 7 rRNAs (including 1 complete 5S rRNA, 1 complete 16S rRNA, 2 partial 5S rRNAs, 3 partial 23S rRNAs), 48 tRNAs, and 4 noncoding RNAs (ncRNAs). The draft genome sequence of *Rhodomicrobium* sp. strain Az07 contains the genes responsible for osmotic adaptation (alpha,alpha-trehalose-phosphate synthase [UDP-forming] [GenBank protein accession number MBT3070931.1], trehalose-6-phosphate synthase [MBT3071046.1], trehalose-phosphatase [MBT3070929.1]), as well as a gene for the sulfate and thiosulfate import ATP-binding protein CysA (MBT3069749.1) involved with selenium uptake.

### Data availability.

The genome sequence has been deposited at NCBI GenBank and is available under accession number JAHFZG000000000.1. The raw sequencing reads are available in the NCBI SRA under accession number SRR14651681.
